# A scaling law for predicting urban trees canopy cooling efficiency

**DOI:** 10.1073/pnas.2401210121

**Published:** 2024-11-04

**Authors:** Jia Wang, Weiqi Zhou, Steward T. A. Pickett, Yuguo Qian

**Affiliations:** ^a^State Key Laboratory of Urban and Regional Ecology, Research Center for Eco-Environmental Sciences, Chinese Academy of Sciences, Beijing 100085, China; ^b^University of Chinese Academy of Sciences, Beijing 100049, China; ^c^Beijing Urban Ecosystem Research Station, Research Center for Eco-Environmental Sciences, Chinese Academy of Sciences, Beijing 100085, China; ^d^Cary Institute of Ecosystem Studies, Millbrook, NY 12545

**Keywords:** urban heat island, cooling efficiency, urban tree canopy (UTC), nature-based solution

## Abstract

Urban heat mitigation is a pressing concern for cities. Intense urban heat poses a threat to human health and urban sustainability. Tree planting is one of the most widely employed nature-based heat mitigation methods worldwide. Therefore, city policy makers require knowledge of how much temperature will be reduced by increasing urban tree canopy (UTC). Cooling efficiency (CE), which was been proposed to quantify the magnitude of temperature reduction associated with a 1% increase in UTC, has been primarily investigated at smaller scales previously. However, such small-scale results cannot be used to develop policy at the whole-city scale. This study developed a method that reveals the scaling relations of CE so as to predict its effects at the city scale. CE was found to follow the form of a power law as spatial scale increased from the small analytical units through intermediate size units up to the extent of a whole city. The power law form appeared consistently across cities with different climate backgrounds during summer daylight hours. Furthermore, the power law form was robust within cities under different summer weather conditions. The power-law scaling approach can thus be used to predict CE at the whole-city scale, providing a useful tool for managers to set UTC goals to mitigate extreme urban heat.

## Significance

Many cities seek to alleviate extreme heat via planting trees. However, the cooling achieved by such programs is debated because previous analyses address scales much smaller than the whole-city scale on which planners operate. To fill this gap, we conducted a scaling analysis of cooling efficiency (CE)—the temperature reduction associated with 1% of increasing urban tree canopy (UTC)—to predict whole-city CE. Results show that CE increased with enlarging spatial scales in a convex power-law form. The power law was consistently found in multiple cities with different climate contexts and was also robust under different summer weather conditions within a city. Power-law scaling of CE can provide a tool for urban planners to set UTC goals for mitigating extreme heat.

**Fig. 1.**
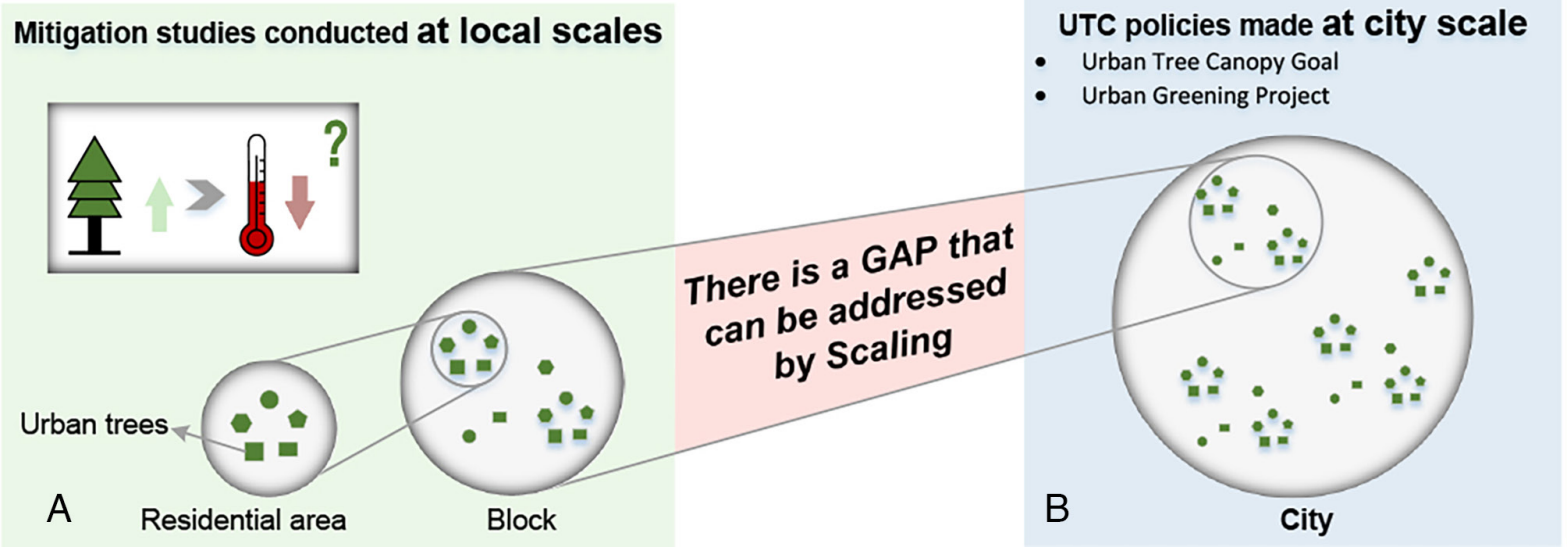
The knowledge gap between scientific understanding of mitigation effects (*A*), and that needed for UTC practice (*B*) can be potentially addressed by the scaling law.

Cities worldwide are experiencing increased extreme heat due to the synergistic effects of global warming and the urban heat island (UHI) effect (1, 2). According to Tuholske et al. (3), global exposure to daily maximum wet bulb temperature of 30 °C increased nearly 200% from 1983 to 2016. Exposure to such extremes is expected to grow with continued global warming and growth of cities (4, 5). To address this challenge, expanding urban tree canopy (UTC) is increasingly used as a nature-based solution for heat mitigation because trees can provide significant cooling effects (6, 7). To set UTC goals, the foremost question managers and decision-makers have asked is, “How much UTC cover does our city need” (8–12)? This question is crucial because cities typically have limited space for greening. Addressing the question is challenging as it must involve social, economic, political, and planning perspectives. Essential to meeting the challenge is rigorous scientific evidence concerning UTC services. One critical gap is the need for managers and decision-makers to understand the magnitude of temperature reduction with different UTC goals at the whole-city scale (13, 14). However, analysis of UTC impacts has mostly been done using spatial units much smaller than a city, such as neighborhoods, resulting in a scale-mismatch between the scientific understanding and that needed in planning practice. Our goal is to bridge this gap by providing planners a tool to quantify how much cooling results from a change in per unit UTC at the scale of entire cities (Fig. 1) (15, 16).

Considerable research has investigated the cooling effects of change in UTC (17, 18). For example, studies have shown that as UTC within neighborhoods increases, temperature significantly decreases (19–21). With one percent of increase in UTC, temperature can decrease 0.04 to 0.57 degrees at these fine scales (22, 23). However, results from these studies cannot answer the question at the whole-city scale because they focused on much smaller scales. While findings at the neighborhood scale can guide planting trees to improve local thermal comfort and livability (19, 20), planners and decision-makers are often concerned about groups of neighborhoods, or larger districts, and ultimately the whole city scale. Whether or not finer-scale results can be extrapolated to the city scale remains unaddressed. Determining whether the cooling effects at the neighborhood scale can be extrapolated to the city scale is a necessary step to address the concern of city managers and planners (16).

Here, we use the well-established cooling efficiency (CE) approach (20, 22) beyond the neighborhood scale. By quantifying the CE at different scales, which is defined as the size of the analytical unit, we test whether there is a statistically robust scaling relationship of CE up to the whole city scale. In particular, we test whether or not the change in CE with the size of analytical unit follows a predictable power-law function. Our tests are inspired by the widely observed power-law scaling relationship in previous studies of biology, ecology, and urban science (24–28). Our research aims to determine whether or not a power law function is useful for extrapolating to the whole city scale. In addition, we investigate the consistency and robustness of these scaling relations under different climate backgrounds and summer weather conditions. Exploring the scaling relationship that links CE and a range of scales is critical to understanding the temperature reduction of urban tree planting and could be harnessed to advance cities’ UTC initiatives.

## Scaling of CE

Scaling as a tool for revealing underlying patterns and processes has been instrumental in understanding issues across the entire scale of systems. Power-law scaling is widely observed in biology, ecology, and urban science studies (24–28). CE—the magnitude of the temperature reduction resulting from the increase of 1% of UTC—can quantify the potential cooling capacity of urban tree planting (19, 22). At each scale, defined as the size of the analytical unit, we measured CE using the absolute values of coefficients estimated from the regression analysis between the percent cover of UTC (Ptree) and land surface temperature (LST). In this study, we plotted the size of the analytical unit against CE and estimated the fit of this relationship to the power-law function, expressed as Eq. **1** (24–29),

[1] $$Q_{S}=kS^{\beta },$$

where S is the size of analytical units, $Q_{S}$ is the CE value, k is the normalization constant, and β is the scaling exponent which quantifies the rate of scaling (28, 29).

## Results

### CE of UTC Scales Following a Power Law.

The CE of UTC in summer daytimes follows a power law relation with the size of the analytical unit S:

[2] $$\mathrm{CE}=(0.057\pm 0.047)S^{\left(0.165\pm 0.106\right)}.$$

**Fig. 2.**
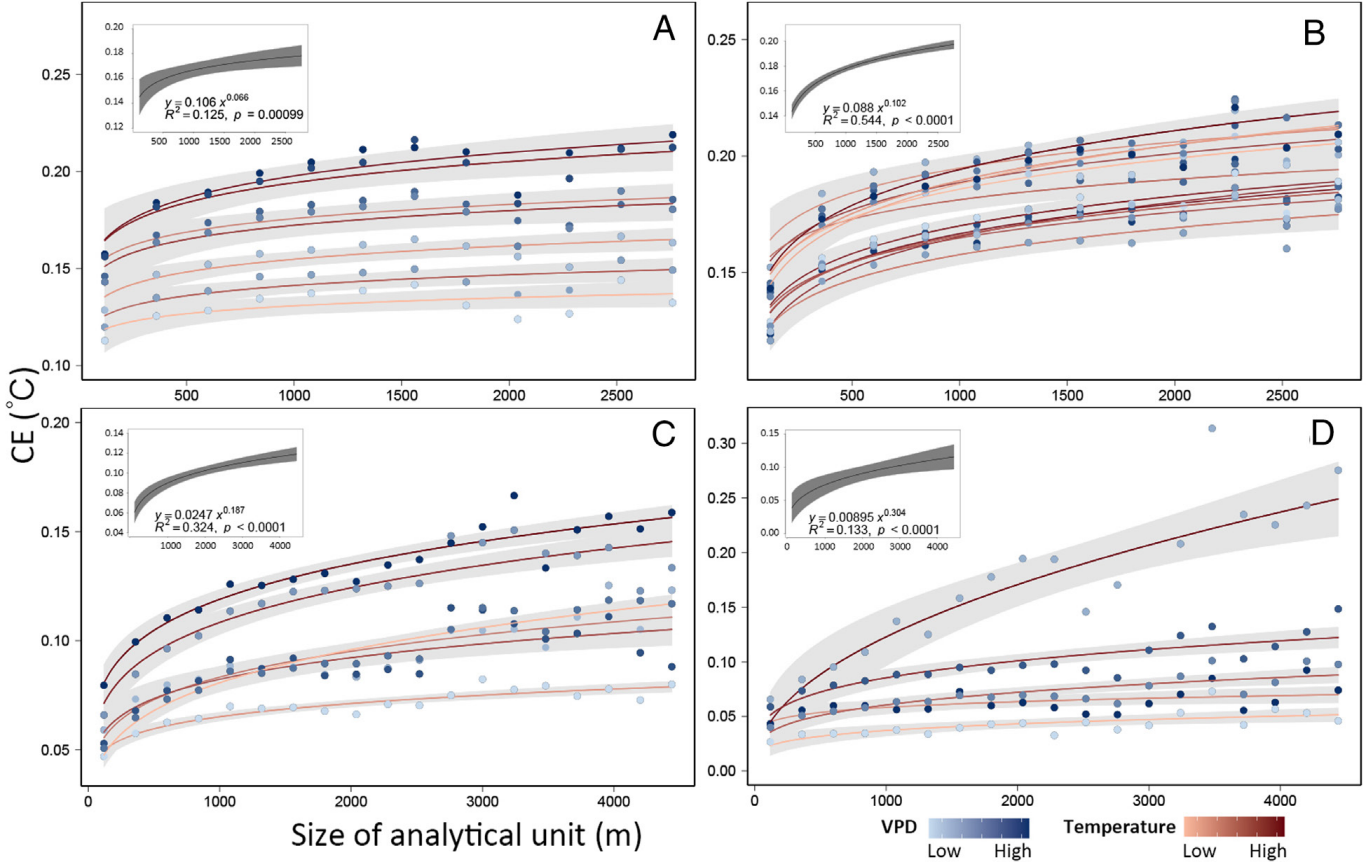
The power-law of CE with increasing the size of the analytical unit in Sacramento (*A*), Baltimore (*B*), Beijing (*C*), and Shenzhen (*D*). The color of solid lines indicates the change in air temperature and that of points indicates the change in vapor pressure deficit (VPD) on those summer sample days. The gray shades of 90% denote 95% CI of the power-law fittings. The solid black lines, shown as insets in the *Upper-Left* corner of each panel, represent the averaged power-law fittings for each city, and the gray shades of 50% denote 95% CI of the fittings.

In particular, CE scaled sublinearly, with the scaling exponent being lower than 1, indicating that CE initially increased sharply with the increase of the size of the analytical unit S, but became relatively stable when the size of the analytical unit was large (Fig. 2 and *SI Appendix*, Fig. S1 and Table S1). The power law as a kind of function was consistent among the four cities, which represent different climatic conditions. Furthermore, it was robust to different summer daytime weather conditions within each city (Fig. 2). Therefore, the power law relationship between CE and the size of analytical units suggested that the CE quantified at smaller scales can be used to predict that at the city scale.

The determination coefficient, R^2^ also tended to increase with the increase of the size of analytical units, suggesting that an increased proportion of the variations of LST was explained by UTC cover when scaling up. Similar to change in CE, the increasing rate of R^2^ at larger scales would be smaller than that at smaller scales (*SI Appendix*, Fig. S2 and Table S2).

### The Power Law Scaling Is Affected by Climatic Conditions.

Although the general pattern of scaling of CE in multiple cities and dates was similar, the parameter of the power law varied across cities and within-city across summer days. The scaling rate—as suggested by the scaling exponent in the power law—varied across the cities having different climatic conditions. CE scaled at low rates in arid cities (Fig. 2). For example, the scaling exponent in Sacramento was 0.066, significantly smaller than that of 0.102 in Baltimore (*P* < 0.01) (Fig. 2 *A* and *B*). As a result, CE increased by 0.03 °C in Sacramento, slower than the 0.06 °C in Baltimore, while shifting from the size of 120 m (i.e., a residential area scale) to the size of 2,760 m (i.e., a census unit scale) (*SI Appendix*, Table S3).

Meanwhile, at the within-city scale, the scaling rate was affected by weather conditions significantly in Sacramento but insignificantly in other cities. In Sacramento, for example, the scaling exponent increased with air temperature significantly in a linear way (*P* < 0.01) (Table 1). Under the extreme day (e.g., mean temperature higher than 24 °C), the quadratic regression, rather than the power law, can produce an even better statistical fit (*SI Appendix*, Fig. S3 and Table S4).

**Table 1.** The relationship between air temperature (°C), wind speed (Wind, m s^−1^), relative humidity (%), VPD (hpa), and scaling exponent (i.e., β in Eq. **1**) in each city

City	Temperature (°C)	Wind (m s^−1^)	Humidity (%)	VPD (hpa)
Sacramento	0.96**	−0.92**	−0.78*	0.91**
Baltimore	0.29	0.03	0.05	0.13
Beijing	−0.19	0.10	−0.74	0.55
Shenzhen	0.76	0.58	0.96**	−0.60

^*^P < 0.05 (two-tailed).

^**^P < 0.01.

## Discussion

### The Mechanism of Power-Law Scaling of CE.

This study is the first to document scale-dependence of the cooling effect of urban trees in the form of a power-law. CE refers to the temperature reduction achieved by increasing UTC by 1%, replacing other surfaces. Increasing UTC will change surface properties and affect local temperature. The mechanisms include changing albedo, which affects solar radiation; changing surface roughness, which affects heat advection; and increasing evapotranspiration, which reduces local temperature. We argue that the change in albedo, which is related to the species composition and canopy structure of UTC, and surface roughness, which is related to the structure of UTC and its interaction with other 3D structures, would be scale-independent. However, the change in evapotranspiration may be scale-dependent because it is significantly related to the size of tree patches (30, 31). As the patch size increases, the evapotranspiration rate first increases and then becomes relatively stable after reaching a certain size of urban tree patch in summer daytime (30–32). Therefore, changes in CE with the change in size of analytical unit is likely related to the scale-dependency of evapotranspiration. However, this warrants further research.

While the power-law distribution has a similar form across different cities, the scaling rate was lower in Sacramento. This phenomenon can be attributed to the fragmented nature of the landscape and very dry summers of the mediterranean climate in Sacramento. Sacramento is characterized by small-sized UTC patches, instead of large forest patches found in the other cities (19). We would expect these small-sized patches in Sacramento to be particularly sensitive to boundary layer effects. This could potentially lead to the relatively small increase in the CE with the increase in size of the analytical unit. This is a topic for future research. Additionally, because the spatial configuration of UTC can affect the cooling effect (19), considering spatial configuration and its scaling also warrants future research.

The variation in LST explained by percent cover of tree canopy (i.e., R^2^) increases with the size of analytical unit. This result is consistent with previous studies (33, 34), which show that bivariate relationships tend to be stronger with the increase of the size of the analytical unit or the decreasing spatial resolution of areal data. The increase in R^2^ is likely due to the statistical “smoothing effect” with the increase of the size of the analytical unit (35). Additionally, the increase in R^2^ might be also related to the scale-dependency of evapotranspiration described above, which warrants further research.

### The Implications of Scaling Relations of CE.

Power-law relationships could help to predict the cooing benefit, i.e., the mitigation magnitude provided by a given amount of new UTC planted at the whole-city scale. Predicting cooling benefits is essential for policy making, for example, setting urban heat mitigation goals and milestones for increasing UTC. We take Baltimore as an example. Our research predicted that a LST reduction of 0.23 °C (0.21 to 0.27) could be achieved if 1% of UTC were added to the city, implying that a goal of increasing UTC by 6.39% (5.62% to 7.21%) could achieve 1.5 °C of temperature reduction (Fig. 2 and *SI Appendix*, Table S1). This prediction is based on the consistent and robust power-law scaling relations of CE found here.

The power law relationships just discussed are found for Beijing, Shenzhen, and Baltimore for all the summer days sampled. Thus, the power law is a general pattern in all cities for all days in the three cities. Even in the arid city of Sacramento, the power law describes the CE relationship on most days with a mean temperature less than 24 °C (*SI Appendix*, Table S1). Adding the extreme day with the highest mean temperature to the analysis, we found the quadratic regression to produce a better statistical fit (*SI Appendix*, Table S4). We expect that this is due to the fundamental physiological limitation of evapotranspiration of trees under extreme heat and drought.

Due to general global warming and drying (36), the air temperature and VPD in urban areas would rise in the future, especially in already hot and dry cities, like Sacramento. Consequently, small values of CE were predicted at the whole city scale on extreme hot days. Thus, the temperature reduction achieved while reaching UTC goals today would be much higher than in a hypothetical but probable hotter and drier future (22, 37). That is, achieving 7.5% increase in UTC- goals in Sacramento could meet 1.5 °C of temperature reduction today (Fig. 2 and *SI Appendix*, Table S1), but larger UTC increases would be required to achieve the same target under future conditions (*SI Appendix*, Fig. S3 and Table S4). This implies that with global climate change, the cooling capacity of UTC will be challenged, and so will mitigation policies.

Here, we used LST to investigate the scaling law of CE to UTC. Although air temperature is the factor that humans directly perceive, and it is the stated concern in urban heat mitigation initiatives (38, 39), LST is a valuable parameter. LST has been widely used as a proxy for urban temperature due to its high spatial resolution and strong correlation with air temperature (40, 41), as well as with health risks (42). Therefore, future research using air temperature to investigate scaling is highly desirable, as it could shed light on the scaling of CE when there are sufficient air temperature data. In this study, we demonstrated that the power law provides a robust relationship for the scaling of CE. Urban planners and policymakers can use this relationship to establish UTC goals for heat mitigation and adaptation.

## Materials and Methods

### Summary.

The research employed three main methodological steps (*SI Appendix*, Fig. S4): 1) We first defined different scales by creating regular grids with different sizes as units of analysis, ranging from 1 × 1 pixel (120 m × 120 m), 3 × 3 pixels (360 m × 360 m), 5 × 5 pixels (600 m × 600 m), to a maximum of 23 × 23 pixels (2,760 m × 2,760 m) for Baltimore and Sacramento, and a maximum of 37 × 37 pixels (4,440 m × 4,440 m) for Beijing and Shenzhen. The largest analytical units depended on the sizes of the cities (19, 43). We extracted the percent cover of UTC (Ptree) from high-resolution remote sensing images and averaged LST from Landsat thermal bands for each analytical unit of all the different scales (*SI Appendix*, Fig. S4 *A* and *B*). We noted that the scaling approach defined here is different from the one widely used in previous studies (e.g., ref. 44), which increases the size of the spatial extent but with a fixed size of analytical unit (*SI Appendix*, Fig. S5). 2) We then calculated the CE by running the ordinary least squares (OLS) regression model of LST and Ptree for each size of analytical unit (*SI Appendix*, Fig. S4*C*). 3) We finally investigated the relationship between CE and the size of the analytical unit using a power-law function to reveal the scaling of CE (*SI Appendix*, Fig. S4*D*). We employed other fitting techniques (i.e., quadratic regression model) with higher fitness if the power function fit is insignificant (*P* > 0.01). In addition, we investigated the consistency and robustness of this scaling relation under different climate backgrounds and weather conditions.

### Study Areas.

We investigated four cities: Beijing and Shenzhen in China and Baltimore and Sacramento in the United States. Those four cities, located in very different biomes, have different climate contexts. Specifically, Shenzhen, built in a biome dominated by tropical and subtropical moist broadleaf forests, has hot and rainy summers (Köppen: *Cwa*), while Sacramento, located in the grassland biome, has hot and dry summers (Köppen: *Csa*). Beijing and Baltimore, belonging to the biome dominated by temperate broadleaf and mixed forests, have hot and humid summers (Köppen: *Dwa, and Cfa, respectively*). The four cities are located in typical biomes (45) and climate types (46), which makes them representative of diverse urban conditions. We focused on the areas within the city limit for Baltimore and Sacramento, that is 239 km^2^ and 259 km^2^, respectively, and the main urban areas for Beijing and Shenzhen that cover 666 km^2^ and 968 km^2^, respectively (47, 48).

### Data.

We mapped the UTC based on high-resolution imagery using an object-based classification approach (49, 50). The high-resolution image data included the 1 m resolution NAIP (National Agricultural Inventory Program) 4-band color-infrared aerial imagery acquired in 2007 for Baltimore and 2010 for Sacramento, and the 1.5 m Pleiades imagery acquired in 2015 for Beijing and 2016 for Shenzhen.

We derived LST from the thermal infrared (TIR) bands of Landsat images that were collected on clear, sunny summer days (*SI Appendix*, Table S1). To increase sufficient sample days to calculate CE, we collected Landsat images 2 y before and after the year the high-resolution imagery was obtained because we assumed that UTC would not change significantly in the short term. LST data were calculated following the methods detailed in Zhou et al. (19).

## Supplementary Material

Appendix 01 (PDF)

## Data Availability

Original data have been deposited in Figshare (51) (https://doi.org/10.6084/m9.figshare.25020404). All other data are included in the manuscript and/or *SI Appendix*.
