# Supplementary material for: A scaling law for predicting urban trees canopy cooling efficiency
Source: Proc Natl Acad Sci U S A. 2024 Nov 4;121(46):e2401210121. doi: 10.1073/pnas.2401210121 (PMC11572964; doi:10.1073/pnas.2401210121)
Supplement: Supplementary file 1 — Appendix 01 (PDF) [file pnas.2401210121.sapp.pdf]

PNAS

Supporting Information for

A scaling law for predicting urban tree canopy cooling efficiency

Jia Wang [a], Weiqi Zhou [a, b, c, *], Steward T.A. Pickett [d, *], Yuguo Qian [a]

[a] State Key Laboratory of Urban and Regional Ecology, Research Center for Eco-Environmental Sciences, Chinese Academy of Sciences, No. 18 Shuangqing Road, Beijing 100085, China

[b] University of Chinese Academy of Sciences, No. 19A Yuquan Road, Beijing 100049, China

[c] Beijing Urban Ecosystem Research Station, Research Center for Eco-Environmental Sciences, Chinese Academy of Sciences, No. 18 Shuangqing Road, Beijing 100085, China

[d] Cary Institute of Ecosystem Studies, Millbrook, New York 12545, USA

[*] Corresponding author: Weiqi Zhou

Email: wzhou@rcees.ac.cn

[*] Corresponding author: Steward T.A. Pickett

Email: picketts@caryinstitute.org

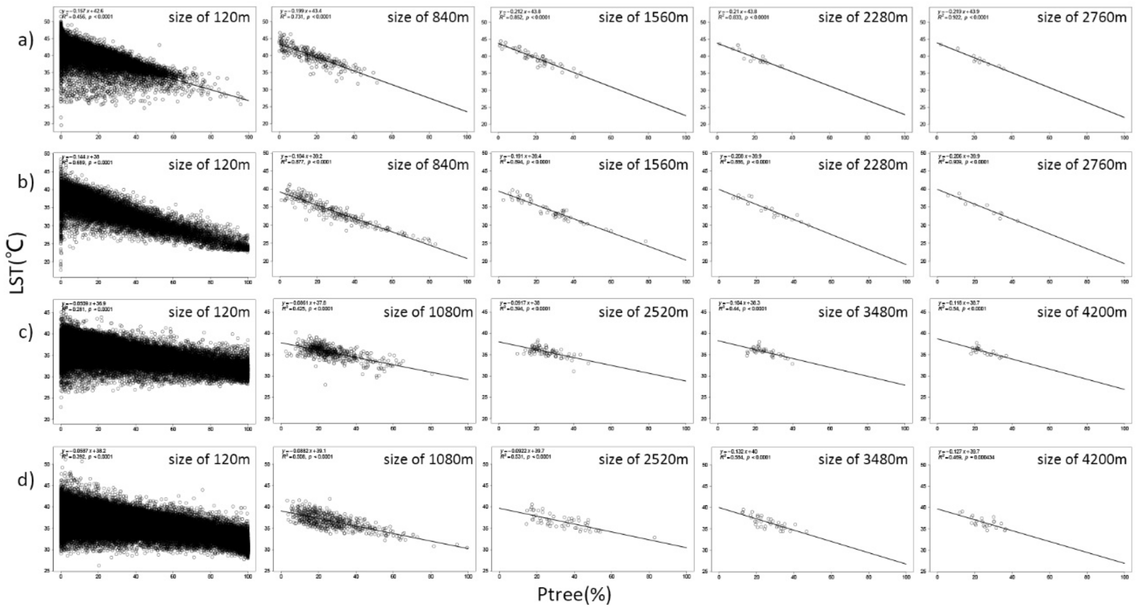

Figure S1. Illustration of scalograms of LST (˚C) and Ptree (%) at sample sizes of the analytical unit in Sacramento on 23 Jul. 2008 (Panel a), Baltimore on 11 Aug. 2007 (Panel b), Beijing on 17 Aug. 2019 (Panel c), and Shenzhen on 18 Sept. 2016 (Panel d). There were a series of scalograms due to the multiple sizes of analytical units and days for the four cities. Here, we have chosen five scalograms for each city as an example.

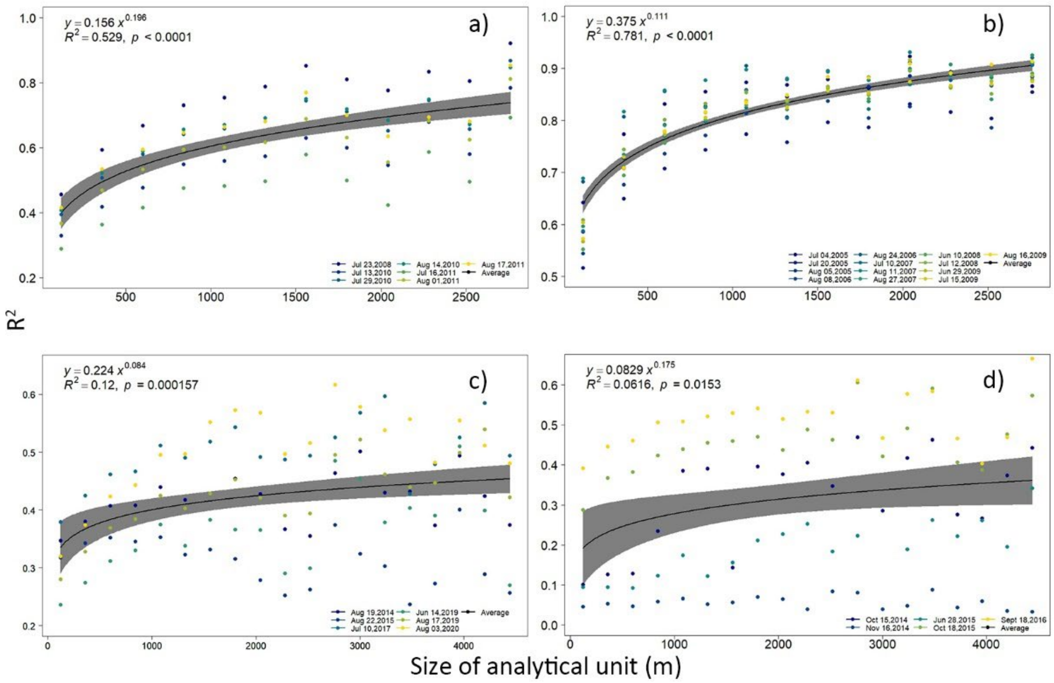

Figure S2. Scalograms of R2 with respect to increasing spatial scales (i.e. the sizes of analysis units or grain sizes) in Sacramento (Panel a), Baltimore (Panel b), Beijing (Panel c), and Shenzhen (Panel d). The black solid lines were fitted by loess methods, and the shades denote 95% confidence intervals.

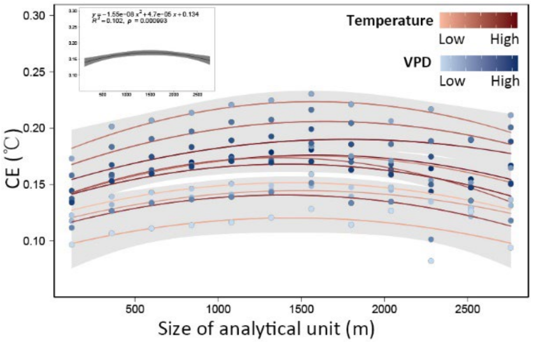

Figure S3. Patterns of cooling efficiency with respect to increasing the size of the analytical unit for several days in Sacramento. The solid lines with different colors represent scaling relations on different days, and gray shades of 90% denote 95% confidence intervals of the individual days. The black solid line with a gray shade of 50% represents the total fitting. The lines were fitted by quadratic regression model (i.e. second order polynomial regression model). The form of the quadratic regression model is $\boldsymbol{Q_S}$=a*S^2+b*S+c, where $\boldsymbol{Q_S}$ is the cooling efficiency in this study, S is the the size of the analytical unit, a, b, and c are coefficients.

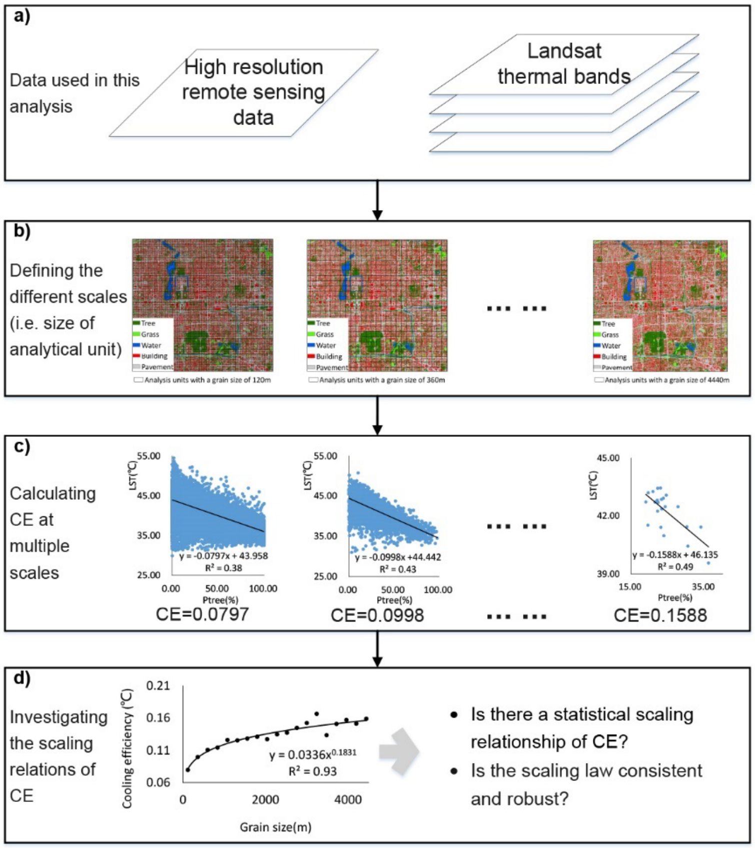

Figure S4. The workflow of this study.

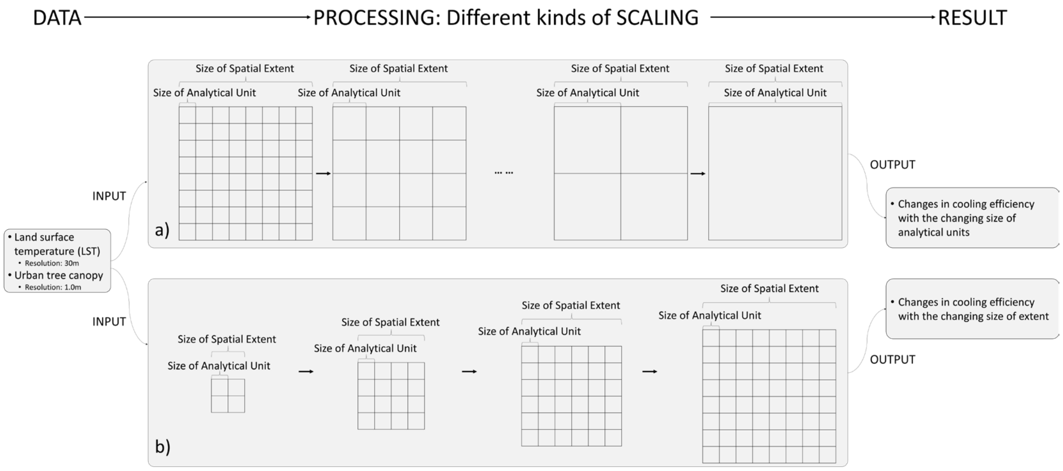

Figure S5. Panel a shows the scaling approach used in this study, with changing size of analytical unit but fixed extent. In contrast, the other widely used scaling approach, showed in panel b, has a fixed size of analytical unit but varied extent.

Table S1. Parameters of the power-law fittings (i.e. equation 1) in multiple days and cities. Shaded rows present weather conditions of these sample days.

City	Day	k	β	R^2	P	Weather condition		
						Air temperature (℃)	Humidity (%)	Wind speed (m/s)
Sacramento	July 23, 2008	0.110	0.085	0.770	**	24.28	45.83	1.78
	July 13, 2010	0.097	0.055	0.605	**	20.57	58.03	4.68
	July 29, 2010	0.117	0.059	0.652	**	23.19	5 5.86	2.28
	August 14, 2010	0.100	0.063	0.718	**	26.96	50.79	2.49
	July 16, 2011	0.096	0.046	0.400	*	21.46	48.98	3.45
	August 01, 2011	0.112	0.062	0.564	**	23.2	48.59	1.78
	August 17, 2011	0.113	0.078	0.676	**	23.48	44.96	2.35
Baltimore	July 04, 2005	0.111	0.081	0.773	**	24.67	62.26	1.9
	July 20, 2005	0.070	0.124	0.896	**	28.5	59.73	2.57
	August 05, 2005	0.080	0.107	0.872	**	29.67	64.29	3.19
	August 08, 2006	0.100	0.092	0.717	**	26.94	58.37	2.67
	August 24, 2006	0.113	0.068	0.732	**	26.17	57.87	1.9

	July 10, 2007	0.082	0.105	0.897	**	28.92	78.06	0.98
	August 11, 2007	0.094	0.099	0.930	**	23.83	66.98	3.91
	August 27, 2007	0.078	0.102	0.846	**	26.14	64.65	2.21
	June 10, 2008	0.085	0.119	0.940	**	30.39	69.96	1.39
	July 12, 2008	0.086	0.094	0.874	**	26.89	65.01	1.9
	June 29, 2009	0.080	0.123	0.921	**	24.33	57.86	2.44
	July 15, 2009	0.087	0.112	0.904	**	24.67	49.12	1.98
	August 16, 2009	0.081	0.104	0.897	**	26.61	68.49	0.93
Beijing	August 19, 2014	0.027	0.128	0.865	**	26.85	64.2	1.71
	August 22, 2015	0.022	0.191	0.791	**	27.08	53.41	2.01
	July 10, 2017	0.033	0.184	0.902	**	30.52	50.68	1.51
	June 14, 2019	0.027	0.164	0.674	**	28.42	46.66	1.63
	August 17, 2019	0.015	0.248	0.938	**	25.72	39.41	1.84
	August 03, 2020	0.028	0.197	0.860	**	30.03	58.55	1.15
Shenzhen	October 15, 2014	0.025	0.122	0.319	*	24.68	54.84	1.54

November 16, 2014	0.008	0.221	0.494	**	22.88	64.41	1.48
June 28, 2015	0.005	0.475	0.718	**	30.66	76.6	2.04
October 18, 2015	0.011	0.251	0.728	**	25.24	60.92	0.96
September 18, 2016	0.016	0.241	0.734	**	28.76	64.5	1.22

Table S2. The statistics of R^2 at multiple scales for the four cities. Shaded rows are standard deviations.

City	Means and standard deviations of R^2 at multiple scales																		
	120	360	600	840	1080	1320	1560	1800	2040	2280	2520	2760	3000	3240	3480	3720	3960	4200	4440
Sacramento	0.3631	0.4681	0.5340	0.5920	0.6118	0.6300	0.7102	0.6672	0.6138	0.6688	0.6021	0.7838							
	0.0777	0.1018	0.1089	0.1156	0.1229	0.1248	0.1303	0.1372	0.1374	0.1696	0.1149	0.1533							
Baltimore	0.5962	0.7292	0.7870	0.8189	0.8460	0.8345	0.8630	0.8474	0.8900	0.8769	0.8609	0.8940							
	0.0507	0.0479	0.0433	0.0381	0.0349	0.0335	0.0256	0.0293	0.0325	0.0228	0.0337	0.0222							
Beijing	0.3135	0.3542	0.3876	0.3965	0.4337	0.4117	0.4405	0.4509	0.4257	0.3808	0.3868	0.4937	0.4915	0.4515	0.4164	0.4078	0.4976	0.4583	0.3830
	0.0502	0.0517	0.0540	0.0538	0.0633	0.0733	0.0825	0.0989	0.0999	0.0998	0.1023	0.0791	0.0938	0.1063	0.1033	0.0798	0.0523	0.1084	0.1021
Shenzhen	0.1669	0.1980	0.2016	0.2446	0.2850	0.2754	0.2409	0.3043	0.2883	0.3520	0.3278	0.4047	0.3227	0.3493	0.3994	0.2940	0.2836	0.3248	0.4091
	0.1393	0.1656	0.1741	0.1818	0.1823	0.2035	0.2009	0.1920	0.1835	0.1809	0.1677	0.2128	0.1662	0.1968	0.1956	0.1507	0.1244	0.1745	0.2191

Table S3. The statistics of cooling efficiency (CE) at multiple sizes of the analytical unit for the four cities. Shaded rows are standard deviations.

City	Means and standard deviations of CE at multiple scales																		
	120	360	600	840	1080	1320	1560	1800	2040	2280	2520	2760	3000	3240	3480	3720	3960	4200	4440
Sacramento	0.1352	0.1549	0.1600	0.1659	0.1704	0.1726	0.1788	0.1690	0.1608	0.1562	0.1627	0.1643	-	-	-	-	-	-	-
	0.0195	0.0245	0.0252	0.0265	0.0281	0.0286	0.0291	0.0300	0.0224	0.0350	0.0297	0.0349	-	-	-	-	-	-	-
Baltimore	0.1354	0.1644	0.1732	0.1776	0.1804	0.1859	0.1878	0.1873	0.1873	0.2033	0.1855	0.1943	-	-	-	-	-	-	-
	0.0107	0.0125	0.0131	0.0134	0.0138	0.0141	0.0140	0.0141	0.0140	0.0157	0.0164	0.0130	-	-	-	-	-	-	-
Beijing	0.0593	0.0746	0.0833	0.0870	0.0959	0.0951	0.0979	0.0966	0.0958	0.0997	0.1003	0.1155	0.1189	0.1203	0.1091	0.1155	0.1221	0.1109	0.1168
	0.0120	0.0152	0.0172	0.0180	0.0203	0.0215	0.0227	0.0247	0.0243	0.0247	0.0258	0.0275	0.0259	0.0325	0.0231	0.0270	0.0273	0.0267	0.0292
Shenzhen	0.0463	0.0592	0.0644	0.0687	0.0742	0.0708	0.0845	0.0859	0.0912	0.0825	0.0742	0.0742	0.0745	0.0987	0.1273	0.0914	0.0985	0.1135	0.1151
	0.0139	0.0176	0.0208	0.0252	0.0349	0.0318	0.0402	0.0478	0.0536	0.0590	0.0394	0.0503	0.0310	0.0599	0.0948	0.0737	0.0662	0.0688	0.0871

Table S4. Parameters of quadratic regression model fittings for different days in Sacramento. Shaded rows present weather conditions of these sample days.

Day	*a*	*b*	*c*	R2	p	Weather condition		
						Air temperature (℃)	Humidity (%)	Wind speed (m/s)
July 07, 2008	-1.57E-08	4.96E-05	1.37E-01	0.740	**	28.06	42.9	1.77
August 08, 2008	-1.27E-08	3.79E-05	1.16E-01	0.768	**	24.28	45.83	1.78
August 24, 2008	-1.95E-08	6.16E-05	1.75E-01	0.694	**	20.57	58.03	4.68
June 24, 2009	-1.30E-08	4.62E-05	1.49E-01	0.564	*	23.19	5 5.86	2.28
July 10, 2009	-1.59E-08	4.93E-05	1.37E-01	0.691	**	26.96	50.79	2.49
July 26, 2009	-1.64E-08	5.40E-05	1.62E-01	0.627	*	21.46	48.98	3.45
August 27, 2009	-2.01E-08	5.49E-05	1.36E-01	0.678	**	23.2	48.59	1.78
June 27, 2010	-1.58E-08	4.49E-05	1.36E-01	0.733	**	23.48	44.96	2.35
August 30, 2010	-1.38E-08	3.99E-05	1.23E-01	0.752	**	25.37	44.41	2.21
June 14, 2011	-1.48E-08	4.12E-05	1.12E-01	0.458	P=0.063	20.54	54.7	4.72
June 30, 2011	-1.30E-08	3.75E-05	9.34E-02	0.331	P=0.164	20.28	52.91	2.98

63
